# Estimation of Liver Fat by FibroScan in Patients With Nonalcoholic Fatty Liver Disease

**DOI:** 10.7759/cureus.16414

**Published:** 2021-07-15

**Authors:** Rupesh Shrestha, Sudhamshu KC, Pukar Thapa, Arbinda Pokharel, Niyanta Karki, Bikash Jaishi

**Affiliations:** 1 Liver Unit, Bir Hospital, National Academy of Medical Sciences, Kathmandu, NPL

**Keywords:** body mass index, controlled attenuation parameter, fibroscan, liver fat, non-alcoholic fatty liver disease

## Abstract

Background

Assessment of nonalcoholic fatty liver disease (NAFLD) includes estimation of liver fat (steatosis). Controlled attenuation parameter (CAP) value obtained by FibroScan^®^ (Echosens, Paris, France) is an alternative to liver biopsy for diagnosing and estimating steatosis (S). This study aimed to estimate the liver fat by CAP in NAFLD patients.

Methods

An observational cross-sectional study was conducted at the Liver Unit of Bir Hospital, from January 2021 to May 2021 after ethical clearance from the Institutional Review Board of the National Academy of Medical Sciences. A convenient sampling method was used. Data were analyzed with descriptive and inferential statistics involving bivariate and multivariate analysis.

Results

A total of 127 NAFLD patients were enrolled. The mean (±SD) CAP value was 271.53 (±50.69) dB/m. Total cholesterol, triglyceride, and body mass index (BMI) correlated positively (p<0.05) while systolic blood pressure correlated negatively with CAP value (p=0.031). On multivariate analysis, patients with BMI ≥25 kg/m^2^ were found 3.7 times more likely to have CAP ≥291 dB/m (S3, severe steatosis) than those with BMI <25 kg/m^2^ (p=0.048, 95% CI 1.01, 13.50). The mean (±SD) CAP values were 276.19 (±49.93) and 246.60 (±48.50) dB/m among those with BMI ≥25 kg/m^2^ and <25 kg/m^2^, respectively (p=0.016, using independent t-test). CAP steatosis grading correlated positively with both the ultrasound grading (p<0.001) and fibrosis grading by liver stiffness measurement (p=0.004).

Conclusion

In this observational cross-sectional study of NAFLD patients, the mean (±SD) CAP value was 271.53 (±50.69) dB/m, which corresponds to moderate steatosis (S2). Obese NAFLD patients with ≥25 kg/m^2^ were 3.7 times more likely to have severe steatosis (S3) than nonobese NAFLD patients with BMI <25 kg/m^2^.

## Introduction

Nonalcoholic fatty liver disease (NAFLD) is the most common cause of fatty liver (steatosis). It represents infiltration of fat in the liver (more than 5-10% of hepatocytes) without excessive alcohol consumption and other causes of liver disease.

The assessment of NAFLD should also include estimation of liver fat. This helps anticipate future cardiometabolic risk, treatment monitoring, and liver graft assessment in live donor [1-3]. Liver biopsy (LB) is the gold standard for diagnosis and assessment of the severity of steatosis and grading of fibrosis. However, it is an invasive method, difficult to reproduce and usually, patients are not ready for this. Controlled attenuation parameter (CAP) value obtained by FibroScan® (Echosens, Paris, France) is an alternative method for diagnosing and estimating steatosis with good accuracy, lower cost, and without any complications.

This study aimed to estimate the liver fat by CAP in NAFLD patients and to find out the correlation between CAP values and ultrasound grading of steatosis and other co-variates.

## Materials and methods

This was an observational cross-sectional study conducted at the Liver Unit of Bir Hospital, a tertiary care center of Nepal, from January 2021 to May 2021 after ethical clearance from the Institutional Review Board of National Academy of Medical Sciences, Bir Hospital, Kathmandu (reference no. 529/2077/78).

All adults (aged ≥18 years) with a diagnosis of NAFLD by ultrasonography (USG) were included in the study while patients with a history of significant alcohol intake (>20 g/day in females and 30 g/day in males), those using medications that can cause fatty liver, pregnant patients, those with acute or chronic viral hepatitis, and those not providing consent were excluded.

Patients were evaluated for the presence of metabolic syndrome (MetS) as per Modified National Cholesterol Education Program Adult Treatment Panel III 2005 criteria [4]. Waist circumference (WC) was measured at the midpoint between the lowest margin of the least palpable rib and the top of the iliac crest in the standing position. Individuals with BMI ≥25 kg/m^2^ were considered obese. NAFLD patients who were not obese (BMI <25 kg/m^2^ in non-Asians, <23 kg/m^2^ in Asians) were defined as “lean NAFLD.” Blood pressure, both systolic blood pressure (SBP), and diastolic blood pressure (DBP) were recorded in the supine position. Hypertension (HTN) was diagnosed when SBP was ≥130 mmHg and/or DBP ≥85 mmHg [4].

Blood samples were obtained and sent for investigations like fasting blood glucose (FBG), fasting lipid profile that included total cholesterol, triglycerides (TG), high-density lipoprotein (HDL) cholesterol and low-density lipoprotein (LDL) cholesterol, glycated hemoglobin (HbA1c), hemoglobin (Hb), and liver biochemistry that included total bilirubin (TB), direct bilirubin (DB), alanine aminotransferase (ALT), aspartate aminotransferase (AST), alkaline phosphatase (ALP), gamma-glutamyl transferase (GGT), prothrombin time (PT) and international normalized ratio (INR). Dyslipidemia was defined as elevation of total cholesterol ≥200 mg/dL, TG ≥150 mg/dL, or both, or a low HDL cholesterol level (<50 mg/dL in females, <40 mg/dL in males).

FibroScan® was used for the measurement of liver stiffness and CAP via transient elastography. The M probe was used in the first instance so that both liver stiffness measurement (LSM) and CAP could be obtained. The XL probe catering for obese patients was used when the M probe failed. At least 10 valid measurements were obtained in each patient. A success rate of ≥60% (number of validated measurements divided by the total number of measurements) and the ratio of the interquartile range (IQR) of liver stiffness to the median (IQR/MLSM) ≤30% were considered reliable and used for the final analysis [5].

Ultrasonography and FibroScan of each patient were performed by different hepatologists with experience of at least five years. Both operators were blinded by the results. CAP cut-off values indicating liver steatosis (S) were adapted from the study by Kamali et al. as follows: (1) <237 dB/m (S0, no steatosis), (2) 237.0-259.0 dB/m (S1, mild steatosis), (3) 259.0-291.0 dB/m (S2, moderate steatosis), and (4) 291.0-400.0 dB/m (S3, severe steatosis) [6]. The cut-off values for fibrosis (F) were also adopted from the same study as follows: (1) <5.5 kPa (F0, no fibrosis), (2) 5.5-8.0 kPa (F1, mild fibrosis), (3) 8.0-10.0 kPa (F2, moderate fibrosis), (4) 11.0-16.0 kPa (F3, severe fibrosis), and (5) >16.0 kPa (F4, cirrhosis) [6]. 

The data were entered in Microsoft Excel which was then cleaned and checked for any inconsistencies. The data were then coded and exported to SPSS for Windows version 16.0 (Chicago: SPSS Inc.) for analysis. Categorical data were described with frequency and percentage, while mean and standard deviation (SD) were calculated along with minimum and maximum values for presenting continuous data.

Inferential statistics involved bivariate and multivariate analysis. Correlation of the continuous independent variables with CAP values was assessed using Pearson correlation. Similarly, correlation of the steatosis grading by CAP with USG grading and fibrosis grading by LSM was done using Spearman rank correlation. Chi-square test was applied to see the association of CAP with different categorical independent variables, while independent t-test was used to compare the CAP values of different categories. Univariate logistic regression was applied to calculate the odds ratio (OR) and its 95% CI for the independent variables. All the variables with p-value <0.25 in univariate logistic regression were considered for collinearity test before taking for multivariate analysis. Those variables with variation inflation factor (VIF <2) in collinearity test were then taken into multivariate analysis. Multivariate logistic regression was applied to calculated adjusted odds ratio and the confidence interval.

## Results

A total of 127 NAFLD patients were enrolled. While 84.3% of the patients were obese (BMI ≥25 kg/m^2^), the mean (±SD) BMI was 28.41 (±3.83) kg/m^2^. More than half of the patients had HTN (51.2%) and MetS (89.98%) (Table 1).

**Table 1:** Basic socio-demographic and clinical characteristics of patients (n=127) BMI: body mass index, SD: standard deviation

Characteristics		Frequency (Percentage)/Mean ± SD (Min, Max)
Sex (male:female)		86 (67.7%):41 (32.3%)
Age (years)		50.25 ± 9.53 (30, 82)
BMI (kg/m^2^)		28.41 ± 3.83 (20.1, 42.2)
BMI ≥25 (kg/m^2^)		107 (84.3%)
Waist circumference (cm)	Male	101.28 ± 4.46 (82.5, 108.5)
	Female	92.00 ± 5.85 (81.6, 104.8)
	Total	98.29 ± 6.58 (81.6, 108.5)
Diabetes		44 (34.6%)
Hypertension		65 (51.2%)
Other chronic diseases		25 (19.7%)
Dyslipidemia		106 (83.5%)
Metabolic syndrome		113 (88.98%)

Most of the patients had dyslipidemia (83.5%) with mean (±SD) total cholesterol, LDL cholesterol, HDL cholesterol, and TG being 222.25 (±50.67) mg/dL, 120.54 (±29.73) mg/dL, 43.3 (±10.29) mg/dL, and 231.61 (±90.89) mg/dL, respectively. Mean of TB, DB, AST, ALP was normal, but mean (±SD) ALT was high, 57.72 (±36.08) IU/L (Table 2).

**Table 2:** Biochemical parameters of the patients (n=127) LDL: low-density lipoprotein, HDL: high-density lipoprotein, TG: triglyceride, FBG: fasting blood glucose, HbA1c: glycated hemoglobin, Hb: hemoglobin, TB: total bilirubin, DB: direct bilirubin, AST: aspartate aminotransferase, ALT: alanine aminotransferase, ALP: alkaline phosphatase, GGT: gamma-glutamyl transferase, PT: prothrombin time, INR: international normalized ratio, SD: standard deviation

Characteristics	Mean ± SD (Min, Max)
Total cholesterol (mg/dL)	222.25 ± 50.67 (113, 346)
LDL cholesterol (mg/dL)	120.54 ± 29.73 (40, 212)
HDL cholesterol (mg/dL)	43.3 ± 10.29 (21, 82)
TG (mg/dL)	231.61 ± 90.89 (35, 405)
FBG (mg/dL)	123.13 ± 50.81 (78, 452)
HbA1c	6.46 ± 0.85 (5, 8.9)
Hb (g/dL)	14.25 ± 1.63 (10.2, 18.5)
TB (mg/dL)	0.94 ± 0.59 (0.2, 4.1)
DB (mg/dL)	0.25 ± 0.18 (0.01, 1.2)
AST (IU/L)	38.7 ± 18.31 (15, 103)
ALT (IU/L)	57.72 ± 36.08 (16, 201)
ALP (IU/L)	116.51 ± 37.24 (47, 258)
GGT (IU/L)	62.05 ± 25.56 (12, 178)
PT (sec)	13.16 ± 1.39 (10, 17.7)
INR	1.05 ± 0.16 (0.8, 1.51)

The mean (±SD) CAP was 271.53 (±50.69) dB/m, but about 20.5% of the patient diagnosed to have NAFLD by USG had no steatosis (S0) during CAP evaluation by FibroScan (Table 3). The mean (±SD) CAP among lean NAFLD (BMI <23 kg/m^2^) patients was 233.83 (±69.39) dB/m (Table 4).

**Table 3:** Ultrasonography grading of fatty liver and FibroScan findings of the patients (n=127) USG: ultrasonography, CAP: controlled attenuation parameter, LSM: liver stiffness measurement, SD: standard deviation

Characteristics	Categories	Frequency (Percentage)
USG grading of fatty liver	1	50 (39.4%)
	2	52 (40.9%)
	3	25 (19.7%)
Steatosis grading by CAP	S0	26 (20.5%)
	S1	22 (17.3%)
	S2	34 (26.8%)
	S3	45 (35.4%)
CAP (Mean ± SD {Min, Max})		271.53 ± 50.69 (100, 382) dB/m
Fibrosis grading by LSM	F0	76 (59.8%)
	F1	35 (27.6%)
	F2	13 (10.2%)
	F3	3 (2.4%)
	F4	0 (0%)
LSM (Mean ± SD {Min, Max})		5.39 ± 1.87 (2.1, 11.6) kPa

**Table 4:** Mean CAP values according to sex, BMI, and steatosis grade (n=127) BMI: body mass index, CAP: controlled attenuation parameter

Characteristics	Categories	CAP Values (dB/m) Mean ± SD (Min, Max)
Sex	Male	268.51 ± 50.21 (100, 373)
	Female	277.85 ± 51.73 (128, 382)
BMI	<23 kg/m^2^	233.83 ± 69.39 (135, 303)
	≥ 23 kg/m^2^	273.4 ± 49.22 (100, 382)
Steatosis grade	S0	199 ± 36.6 (100, 235)
	S1	248.55 ± 6.17 (239, 258)
	S2	275.47 ± 9.31 (260, 289)
	S3	321.69 ± 23.56 (291, 382)
Total		271.53 ± 50.69 (100, 382)

Table 5 shows Pearson’s correlation coefficient and the p-value for each of those correlations which depict that the total cholesterol level, TG, and BMI were positively correlated with CAP value, and the correlation was statistically significant (p<0.05). Similarly, SBP was found to be negatively correlated with statistically significant p-value. Other variables such as LDL cholesterol, HDL cholesterol, and FBG were positively correlated while age, DBP, WC, and ALT levels were found to be negatively correlated with CAP value; however, those were not statistically significant.

**Table 5:** Correlation of CAP values of FibroScan with other co-variates (n=127) CAP: controlled attenuation parameter, LDL: low-density lipoprotein, HDL: high-density lipoprotein, TG: triglyceride, SBP: systolic blood pressure, DBP: diastolic blood pressure, BMI: body mass index, WC: waist circumference, FBG: fasting blood glucose, ALT: alanine aminotransferase

Covariates	Pearson Correlation	P-value
Age	-0.037	0.680
Total cholesterol	0.234	0.008
LDL cholesterol	0.140	0.115
HDL cholesterol	0.022	0.808
TG	0.227	0.010
SBP	-0.192	0.031
DBP	-0.107	0.231
BMI	0.275	0.002
WC	-0.046	0.604
FBG	0.070	0.432
ALT	-0.007	0.940

On Spearman rank correlation, CAP steatosis grading was correlated positively and significantly with both the USG grading of fatty liver (p<0.001) and fibrosis grading by LSM (p=0.004) (Table 6).

**Table 6:** Correlation of CAP steatosis grading with USG grading and fibrosis grading CAP: controlled attenuation parameter, USG: ultrasonography, LSM: liver stiffness measurement

Grading	Spearman rank correlation	P-value
USG grading of fatty liver	0.623	<0.001
Fibrosis grading by LSM	0.252	0.004

When univariate logistic regression was applied to calculate the odds ratio and its 95% CI for each of the independent variables, those with BMI of ≥25 kg/m^2^ were 3.66 times (95% CI 1.01, 13.26) likely to have CAP ≥291 dB/m (S3) than those with BMI <25 kg/m^2^, and it was statistically significant. Similarly, those with higher TG values were 2.12 times likely to have CAP ≥291 dB/m than those with lower TG values. There were increased odds of having CAP ≥291 dB/m among females, those aged <40 years, diabetic, those with elevated WC and ALT >40 IU/L (Table 7).

**Table 7:** Factors associated with severe steatosis by univariate and multivariate logistic regression HDL: high-density lipoprotein, TG: triglyceride, WC: waist circumference, BMI: body mass index, ALT: alanine aminotransferase, AOR: adjusted odds ratio

Characteristics	OR (95% CI)	p-value	AOR (95% CI)	P-value
Sex (male)	0.80 (0.37, 1.72)	0.559	-	-
Age <40 years	1.70 (0.57, 5.05)	0.337	-	-
Diabetes	1.06 (0.50, 2.28)	0.873	-	-
Hypertension	0.87 (0.42, 1.80)	0.702	-	-
Low HDL (<40 mg/dL in males, <50 mg/dL in females)	0.99 (0.48, 2.06)	0.991	-	-
High TG (≥150 mg/dL)	2.12 (0.83, 5.41)	0.118	2.14 (0.83, 5.54)	0.118
Elevated WC (≥90 cm in males, ≥80 cm in females)	1.67 (0.17, 16.55)	0.661	-	-
BMI ≥25 kg/m^2^	3.66 (1.01, 13.26)	0.048	3.70 (1.01, 13.50)	0.048
ALT >40 IU/L	1.03 (0.47, 2.25)	0.945	-	-

The two variables high TG and BMI were taken to multivariate analysis. BMI ≥25 kg/m^2^ was still statistically significant in multivariate analysis with adjusted OR of 3.70 (95% CI 1.01, 13.50) with p-value of 0.048 implying that those with BMI ≥25 kg/m^2^ were 3.7 times more likely to have higher CAP values (≥291 dB/m) than those with BMI <25 kg/m^2^.

**Figure 1:**
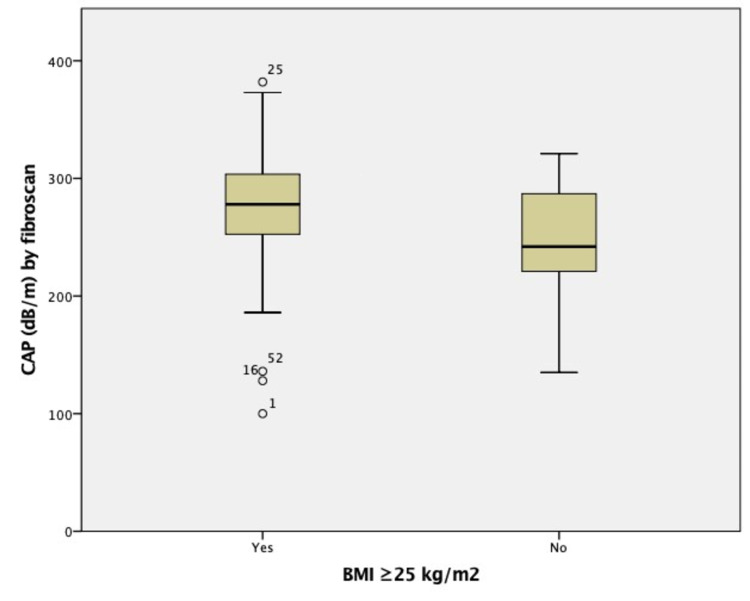
Comparison of CAP values among those with BMI ≥25 kg/m2 and with <25 kg/m2 CAP: controlled attenuation parameter, BMI: body mass index

The mean (±SD) value of CAP was 276.19 (±49.93) dB/m for those with BMI ≥25 kg/m^2^, and it was 246.60 (±48.50) dB/m for those with BMI <25 kg/m^2^, and the difference was statistically significant (p=0.016, using independent t-test) (Figure 1).

## Discussion

Nonalcoholic fatty liver disease represents a major public health challenge. It is associated with type 2 diabetes, MetS, and other cardiovascular risk factors, and may lead to fibrosis, cirrhosis, liver cancer, liver failure requiring liver transplant, and mortality [7-11].

Controlled attenuation parameter is reported to be highly sensitive in detecting low-grade steatosis as fat deposition ≥10%, and its values correlated well with the amount of steatosis assessed by LB in previous studies from different countries [3,12,13-16]. CAP can be performed simultaneously to LSM and in the same liver volume, making possible the simultaneous evaluation of both fibrosis and steatosis and thus consequently enhancing the spectrum of noninvasive methods for the exploration and follow-up of patients with chronic liver disease. In comparison with other modalities, CAP presents the advantage of being non-ionizing, inexpensive, and nonsubject to the operator interpretation. 

The mean (±SD) CAP value of 127 NAFLD patients enrolled in this study was 271.53 (±50.69) dB/m which corresponds to S2 steatosis. Paul et al. also reported the mean CAP value of 278.57 (±49.13) dB/m in NAFLD patients during the first consultation [17]. Interestingly, 26 (20.5%) patients diagnosed to have NAFLD by USG were unable to be diagnosed with fatty liver in accordance with the standard of CAP value by FibroScan. This could be due to the presence of hepatic fibrosis causing increased echogenicity of the liver on USG images, leading to a misdiagnosis of fatty liver. Also, because the mean BMI of patients in this study was 28.41 kg/m^2^ and since for severely obese individuals, the sensitivity and specificity of USG in detecting NAFLD falls to 49% and 75%, respectively, possibly due to image blurring caused by thickening of the abdominal subcutaneous and visceral fat, patients might be misdiagnosed as having fatty liver in USG [18].

However, liver USG is an accurate, reliable tool to detect moderate to severe fatty liver, with sensitivity and specificity of 84.8% and 93.6%, respectively [19]. Also, the CAP value significantly correlates with the USG-based steatosis grading and the median CAP value for each USG-based steatosis grade shows a positive dose-response relationship [20]. This observation is in line with our study also, which showed CAP steatosis grading correlated positively and significantly with the USG grading of fatty liver (p<0.001).

However, the CAP value is susceptible to interference by liver fibrosis in NAFLD [21]. This study also showed that CAP steatosis grading correlated positively and significantly with fibrosis grading (p=0.004). A similar observation has also been reported by Sehgal et al. [22]. To note is that 40.2% of patients in the current study had some degree of fibrosis, measured as LSM by FibroScan and 12.6% had significant fibrosis (F2-F4); findings similar to the other study [23].

CAP values are associated with metabolic disorders [24]. Our study proved the same total cholesterol level, TG, and BMI correlated positively and significantly (p<0.05) with CAP value. These findings are also in line with Mansour et al. and Kwok et al. [23,25].

In our study, the mean (±SD) value of CAP was 276.19 (±49.93) dB/m for those with BMI ≥25 kg/m^2^, and it was 246.60 (±48.50) dB/m for those with BMI <25 kg/m^2^, and the difference was statistically significant (p=0.016). In addition, those with BMI ≥25 kg/m^2^ had 3.7 times (95% CI 1.01, 13.50) more likely to have CAP ≥291 dB/m (S3) than those with BMI <25 kg/m^2^ in multivariate analysis, and it was statistically significant. Shalimar et al. also showed BMI as an independent predictor of CAP even after adjusting for diabetes, serum TG, serum bilirubin, and age in a study done in India [26].

By non-Asian criteria (BMI <25 kg/m^2^), the current study constituted 15.7% lean NAFLD patients, which is comparable to the other study (11.5%) [27]. While by Asian criteria (BMI <23 kg/m^2^), lean NAFLD constituted only 4.7% of total NAFLD patients which is lower than that reported in an Indian report (13.2%) [28]. In the current study, the mean CAP (±SD) of lean NAFLD (BMI <23 kg/m^2^) patients was 233.83 (±69.39) dB/m.

The present study showed that SBP negatively and significantly correlated with CAP value. This finding is in contrast to the common dictum that CAP value, which is an indicator of steatosis, increases with the increase in the number of MetS components including HTN [29,30]. This disparity could be explained by the fact that most of the patients enrolled in this study were referred from general physicians for evaluation of fatty liver and were already getting treatment for comorbid conditions including HTN, and since more than half of the enrolled patients (51.2%) in this study were hypertensive and under medications, this could have generated a contradicting inference of correlation between NAFLD and blood pressure.

## Conclusions

The mean CAP value of NAFLD patients enrolled in this study was 271.53 (±50.69) dB/m that corresponds to moderate steatosis. However, obese NAFLD patients with BMI ≥25 kg/m^2^ were 3.7 times likely to have severe steatosis than non-obese NAFLD patients with BMI <25 kg/m^2^.

In this study, CAP steatosis grading correlated positively and significantly with the USG grading of fatty liver and fibrosis grading by LSM. So, liver fat estimation in NAFLD patients can be done reliably with USG.
